# Elevated frequency and severity of asthma in patients with hiatal hernia: A retrospective study

**DOI:** 10.3892/mi.2024.209

**Published:** 2024-12-02

**Authors:** Michel Abou Khalil, Khalil Hamadeh, Mario Fakhry, Elissa Chebly, Moussa Riachy, Hind Eid, Zeina Aoun Bacha

**Affiliations:** 1 Faculty of Medicine, Saint Joseph University, Beirut 1107-2180, Lebanon; 2 Department of Pulmonary and Critical Care Medicine, Hotel Dieu de France, Saint Joseph University, Beirut 1107-2180, Lebanon

**Keywords:** hiatal hernia, asthma, gastroesophageal reflux disease, PPI, severity

## Abstract

Hiatal Hernia (HH) and gastroesophageal reflux disease (GERD) have been found to be associated with respiratory conditions, such as pulmonary fibrosis. However, their association with asthma remains ambiguous. Thus, the present cross-sectional, retrospective, monocentric study aimed to investigate the prevalence of asthma among patients with HH, evaluate its severity in these patients, and screen for associated respiratory symptoms. Additionally, the present study explored the association between the prevalence of asthma and various parameters, including sex, GERD medications and symptoms. For this purpose, a retrospective study, conducted at one central university medical center from January, 2020 to May, 2023, included patients with HH identified on a computed tomography scan. Patients were contacted and evaluated using structured questionnaires. Asthma-free patients underwent assessment for respiratory symptoms indicative of asthma using a validated questionnaire from the European Community Respiratory Health Survey. In patients with asthma, disease severity was assessed using the Global Initiative for Asthma symptom control criteria. The results revealed that out of 17,374 scans, 1,308 (7.53%) were positive for HH. Among the 453 cases eligible for analysis in the present study, 67 (14.79%) were diagnosed with asthma, of which 28 (41.79%) were diagnosed with uncontrolled asthma. Among the asthma-free patients, 136 (35.23%) reported at least one unspecified respiratory symptom. In the patients with HH, sex and GERD exhibited showed no association with asthma (P=0.07 and P=0.11, respectively). However, patients taking GERD medications exhibited a higher prevalence of asthma (P=0.03). On the whole, the present study demonstrates that the prevalence of asthma in patients with HH appears to be elevated. Hence, an ambivalence arises as regards the presence of a HH potentially associated with poorly controlled asthma and GERD medication potentially exacerbating asthma.

## Introduction

Hiatal hernias (HHs) are a relatively common condition in the general population that occur due to elevated intra-abdominal pressure, leading to the protrusion of the stomach and other abdominal viscera into the mediastinum (1). The diagnosis of gastroesophageal reflux disease (GERD) appears to be associated with symptomatic instances of HHs, with regurgitation and retrosternal burning being typical signs of the condition (2,3). Less common symptoms include dysphagia, chest discomfort, or epigastric pain, along with chronic iron-deficiency anemia (4,5).

Apart from their primary gastrointestinal impacts, HHs have been linked to respiratory symptoms. Various studies with patient-reported outcomes reinforce the widely held belief that surgical intervention positively affects respiratory function in patients with HHs, adding to the evidence supporting the intricate association between HHs and respiratory symptoms (6-8). However, respiratory issues in these patients have been historically overlooked. Previous studies have demonstrated a significant association between idiopathic pulmonary fibrosis and GERD due to the presence of HH, resulting in an increased risk of respiratory-related mortality, particularly among patients with HH and idiopathic pulmonary fibrosis (9,10).

Furthermore, as of early 2023, the incidence of COVID-19 in Lebanon stood at 0.94%, highlighting its ongoing impact on respiratory health worldwide (11). Given the overlap between conditions such as COVID-19 and other respiratory illnesses, it is essential to factor in its potential contribution to respiratory symptoms observed in the general population during this period.

Nevertheless, the connection between HHs and other pulmonary conditions, such as asthma, remains unclear. The present study thus aimed to estimate the prevalence of asthma among patients with radiologically confirmed HH and compare to data in the general population. Additionally, the present study sought to assess the severity of asthma in diagnosed patients using a validated score and determine the frequency of non-specific respiratory symptoms related to asthma in otherwise asthma-free patients.

## Patients and methods

### Study design

The present study was a cross-sectional descriptive epidemiological study with a retrospective data collection scheme, conducted using a computerized database sourced from the Radiology Department of Hotel Dieu de France of Beirut, Lebanon spanning from January, 2020 to May, 2023.

Computed tomography scans, conducted using a 64-slice scanner, were independently analyzed by two radiologists working in tandem. Through a blinded evaluation, the radiologists unanimously agreed on the diagnosis of HH. It is crucial to note that the scans used for HH detection encompassed thoracic, abdominal, thoraco-abdominal and thoraco-abdomino-pelvic scans.

### Selection of patients

**Figure 1:**
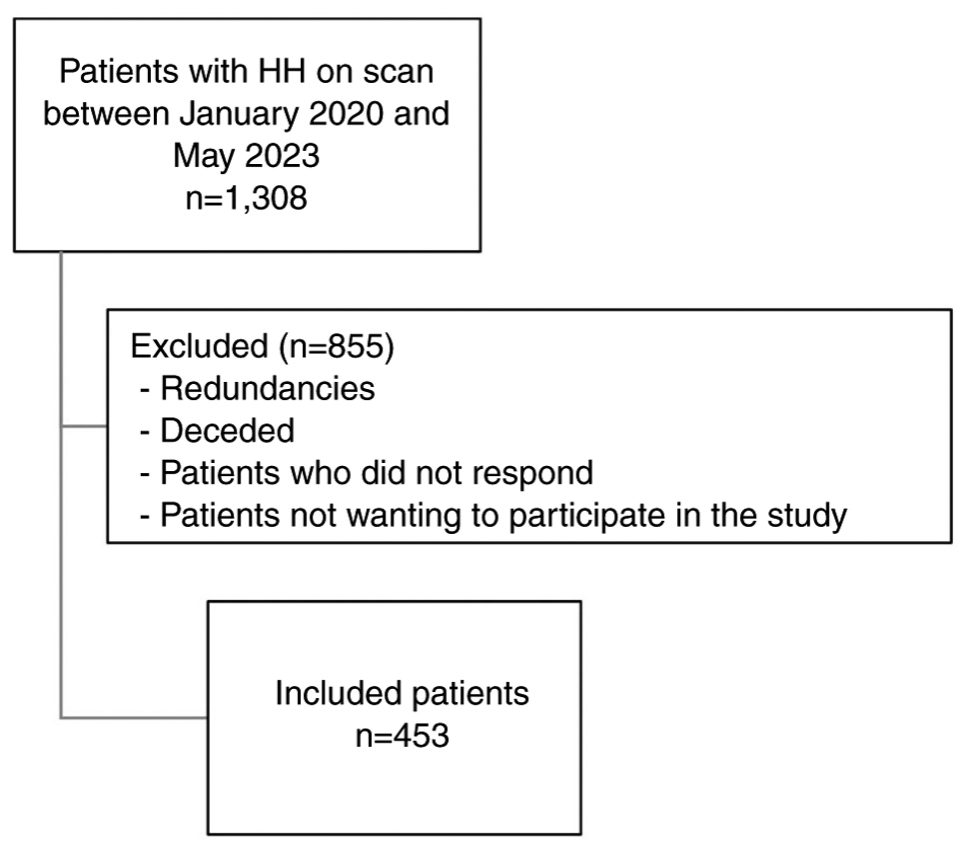
Flowchart of the patient selection used in the present study.

The process of selecting patients for the present study is outlined in Fig. 1. From an initial group of 1,308 patients diagnosed with HH between January, 2020 and May, 2023, a meticulous selection procedure was undertaken to ensure a well-represented study sample. Patient identifiers were retrieved from the hospital system, and consecutive patients were contacted by phone and asked to engage in the study. Notably, 456 patient names were redundant. Additionally, 65 patients passed away during the study period. The eligibility criteria included a clinically and radiologically confirmed diagnosis of HH and a comprehensive respiratory evaluation with a detailed history of asthma symptoms. Patients who did not undergo complete diagnostic workups for both HH and asthma, or those with a recent respiratory infection or who did not fully respond to the questionnaire, were excluded from the study. Among the remaining cohort, 334 patients did not respond to phone calls or declined participation. Ultimately, 453 patients were retained for the analysis.

### Data collection

**Figure 2:**
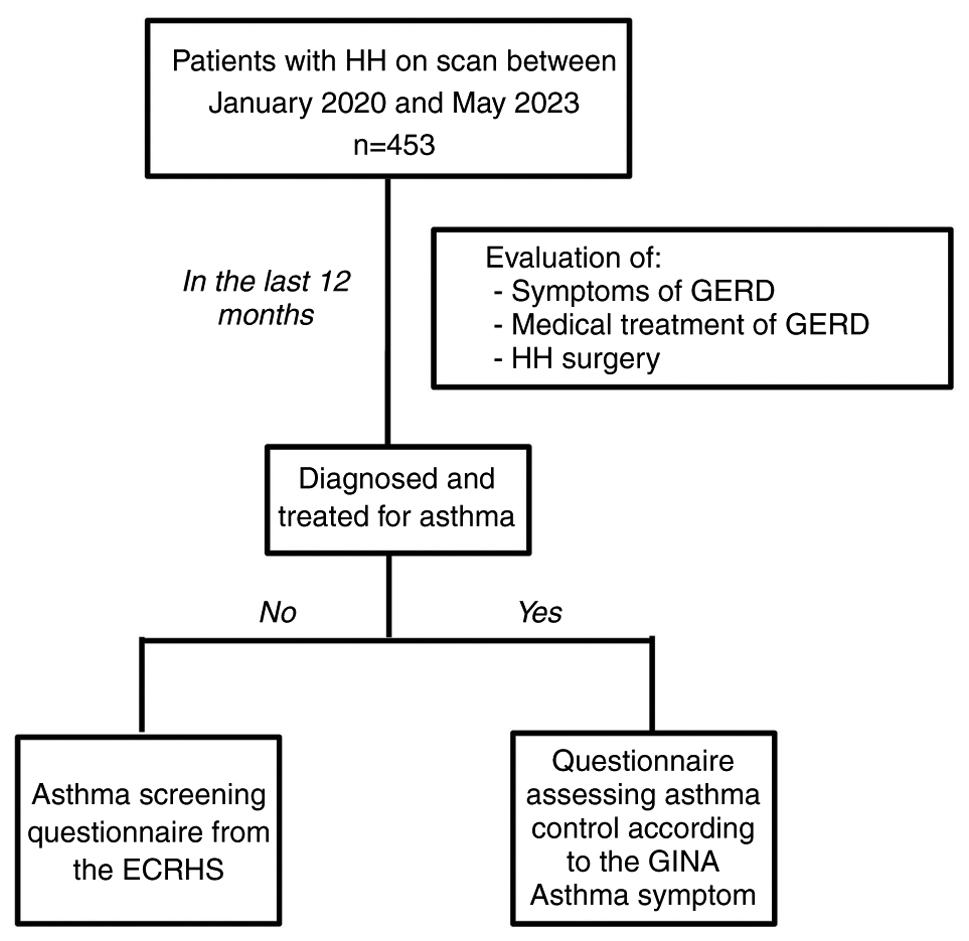
Structured inquiry process and interview strategies used for the participants in the present study. HH, hiatal hernia; GERD, gastroesophageal reflux disease; GINA, Global Initiative for Asthma; ECRHS, European Community Respiratory Health Survey.

Data were retrospectively collected from existing medical records and entered onto a database hosted on Microsoft Office Excel. Patients were also administered a structured questionnaire and were assisted in completing the questionnaire, which remained in English. The interview process, based on patient responses, is summarized in Fig. 2.

The initial segment included inquiries about GERD symptoms, medication use for GERD and a surgical history related to HH (please see ‘eTable I’, Data S1). Screening questions for asthma-associated respiratory symptoms were exclusively addressed to asthma-free patients. These patients were invited to complete a questionnaire adapted from the European Community Respiratory Health Survey (ECRHS), a widely employed tool in epidemiological investigations to quantify respiratory symptoms and screen for asthma (please see ‘eTable II’, Data S1).

Patients diagnosed with and treated for asthma by a pulmonologist were requested to complete the GINA questionnaire assessing the severity of asthma and comprehensively evaluating asthma control in adults, adolescents and children (please see ‘eTable II’, Data S1).

### Objectives

The main objective of the present study was to estimate the prevalence of asthma in patients with radiologically detected HH, and compare it with prevalence data reported in the literature for the general population. Additionally, among patients diagnosed with asthma, disease severity was assessed using a validated score, and among asthma-free patients, the prevalence of non-specific respiratory symptoms related to asthma were determined. The present study also compared the prevalence of asthma among patients with symptomatic HH (reflux symptoms) and asymptomatic HH (no reflux symptoms), and also based on sex and on the use of HH therapy.

### Ethics approval

The present study obtained ethics approval from Hotel Dieu de France's review board [Tfem-2023-8]. The present study received ethics approval in 2022, and patient information was collected retrospectively for the period between 2020 and 2023. The ethics approval allowed for the use of existing patient records up until the final data collection date. Each patient contacted for participation in the study received comprehensive information about the research objectives. Patients were fully aware that their participation in the study was entirely voluntary and that they had full authority to refuse participation. Their confidentiality was strictly protected throughout the study. Patients who agreed to participate provided verbal consent.

### Statistical analysis

In the descriptive approach, counts and percentages were used to estimate the prevalence of asthma among patients with radiologically detected HH. Additionally, frequencies of different symptoms, severity scores, as well as other relevant parameters were calculated. For prevalence comparisons between subgroups, the Chi-squared test was used to assess statistical significance. A P-value <0.05 was considered to indicate a statistically significant difference. All statistical analyses were performed using Microsoft Office Excel 2023.

## Results

A total of 453 patients were included in the analysis. The demographic characteristics of the patients are summarized in Table I. In this comprehensive survey, the frequency of HH was determined across a variety of computed tomography scans, including thoracic, thoraco-abdomino-pelvic and abdominal scans. Over the period spanning from January, 2020 to May, 2023, 1,308 patients out of 17,374 scans examined (7.53%) had radiologically confirmed HH.

### HH and GERD

Initially, the presence of GERD-related symptoms was examined, including retrosternal burning, unexplained cough and the sensation of a lump in the throat. In fact, 279 participants reported experiencing GERD symptoms, accounting for 61.59% of a total of 453 patients. Conversely, 174 participants reported no GERD symptoms, representing 38.41% of the surveyed population.

The use of medications, such as proton pump inhibitors (PPIs) and other reflux treatments for HH was reported and categorized as medications as part of polypharmacy or medications specifically used for symptom management. Among the participating patients, 167 (36.87%) reported taking medications for reasons, such as gastric protection, often as part of a broader polypharmacy regimen, even in the absence of GERD-related symptoms. Additionally, 119 (26.27%) patients were using medications to treat HH-specific symptoms. Another 167 (36.87%) patients were not taking any medications for HH.

Among the 453 patients retained for the analysis, there were 16 instances (3.53%) of surgical intervention for HH repair, notably Nissen fundoplication. The remainder of the patients, [437 (96.47%)], had not undergone any HH-related surgical intervention for HH.

### Concomitant diagnosis of HH and asthma

Among the study population of 453, 67 patients (14.79%) reported a diagnosis of asthma and ongoing management by pulmonologists.

*History of asthma among patients with HH.* Of note, two perspectives were considered when assessing the severity of asthma in the 67 patients. Firstly, the frequency of specific components contributing to asthma symptom control was reported. Specifically, 29 patients (43.28%) reported experiencing daytime asthma symptoms more than twice a week, and another 29 patients reported nocturnal awakenings due to asthma. Half of the patients [34 (50.75%)] used short-acting β-agonist inhalers to relieve their symptoms more than twice a week, and 39 patients (58.21%) reported some form of activity limitation attributed to asthma.

Secondly, the GINA scoring system was used to assess overall asthma control (Table II), with modified descriptors for each score. In this framework, 16 patients (23.88%) had a score of 0, indicating ‘well-controlled’ asthma, 23 (34.33%) had a score of 1 or 2, indicating ‘partially controlled’ symptoms and 28 (41.79%) had a score of 3 or 4, indicating ‘uncontrolled’ asthma symptoms.

*Prevalence of other respiratory symptoms among patients with HH.* Non-specific respiratory symptoms reported by the asthma-free study participants are presented in Table III. The proportion of patients reporting at least one respiratory symptom over the previous 12 months was 35.23% (136 out of 386 participants). The average number of symptoms was 0.6 symptom per subject. No patient reported experiencing all five proposed symptoms during the same period. Finally, within this population of asthma-free patients, the most frequently reported symptom was waking up with a sensation of tightness or shortness of breath (22.54%). Furthermore, 26.68% of this patient population reported nasal allergies, including hay fever.

### Prevalence of asthma in different population subgroups

Subsequently, within the population of patients with HH, the prevalence of asthma was compared based on sex, the presence GERD symptoms, and the use of HH medications (Table IV).

*Based on sex.* Patients with HH and a diagnosis of asthma included 19 males (10.98%) and 48 females (17.14%); the P-value for the difference between males and female was 0.07, suggesting the absence of a statistically significant association between sex and the prevalence of asthma among patients with HH.

*Based on GERD symptoms.* Patients were stratified into two subgroups, based on the presence and absence of GERD symptoms (such as chest burns and cough). Asthma was present among 47 patients with GERD symptoms (16.85%) and among 20 patients with no GERD symptoms (11.49%); the P-value for the difference between those with GERT symptoms and those without was 0.1, indicating the absence of a statistically significant association between the presence of GERD symptoms and a diagnosis of asthma.

*Based on the use of HH medications.* The prevalence of asthma was compared between patient subgroups based on the use of HH medications. Among the patients with HH taking medications (a total of 286 patients), 50 (17.48%) had asthma, while 236 were asthma-free. Among patients reporting no medication use (a total of 167 patients), a lower proportion of patients had asthma [17 (10.18%)], and 150 were asthma-free. The P-value for this difference was 0.03, suggesting a significant association between the use of medications for GERD and the prevalence of asthma.

## Discussion

To the best of our knowledge, the present study is first to examine the prevalence of asthma within a patient population with radiologically confirmed HH. Innovatively, the present study also aimed to assess disease severity in patients with a known diagnosis of asthma, and to conduct early screening for respiratory symptoms in patients with HH and no known respiratory disease. The present study provides a comprehensive perspective on the prevalence, severity and early screening of asthma, and other respiratory conditions in patients with radiologically confirmed HH.

The clinical relevance of HH is well-established, given its implication in GERD, its association with interstitial lung diseases and micro-aspirations (12,13), and its tendency to decrease quality of life (14). However, little is known about the prevalence of HH and its association with asthma. This is, at least in part, due to the fact that asymptomatic individuals are rarely subjected to standard HH tests, such as endoscopy, manometry, or esophago-gastro-duodenal transit.

The present study reported a 7.53% prevalence of radiologically detected HH, including the diaphragmatic hiatus, in computed tomography scans analyzed for 17,374 patients, over the period from January, 2020 to May, 2023. This finding aligns with the results of a study conducted by Rahman *et al* (15), which reported a similar prevalence of 8.8%.

The average age of the patients with HH was 67 years (interquartile range, 63-83), a result consistent with a meta-analysis including data from seven studies (16). That meta-analysis revealed a significant association with an age >50 years and the occurrence of HH. This association may be attributed to the decreased elasticity of the phrenoesophageal ligament, responsible for anchoring the esophagus to the diaphragm and potentially contributing to increased susceptibility to the development of HH.

Furthermore, 280 (61.81%) patients were female, a distribution consistent with the results of another study (17), suggesting a potential female predilection among patients with HH. In the present our study population, 3.53% of the patients with HH had undergone surgical intervention for HH repair, a percentage higher than reported in comparable studies (18). This may be attributed to factors, such as an increased awareness of surgical interventions for HH, evolving surgical techniques, or higher severity of hernias prompting surgical management in this particular cohort.

Collectively, these findings underscore the critical role of HH as a structural contributor to GERD (19,20).

In the present study, it was observed that 67 out of the 453 patients with HH had asthma (14.79%), previously diagnosed and managed clinically. Notably, this prevalence exceeds the global prevalence of asthma in the general population, which was estimated at 3.57% in 2017(21), as well as the reported prevalence of physician-diagnosed asthma in Lebanon, reported at 6.7% (22). The elevated rates of asthma in patients with HH underscores a potentially higher prevalence of asthma among patients with HH, compared to global and national averages. This finding is consistent with that of another study where HH was more frequently observed in patients with asthma rather than asthma-free subjects (62 vs. 34%, P=0.02) (23). This observation is warranted to prompt medical attention and asthma screening in this patient subset.

Several explanations have been proposed to elucidate the association between asthma and HH-induced GERD. Pulmonary hyperinflation is common among patients with asthma. The descent of the diaphragm during pulmonary hyperinflation and increased respiratory effort results in a high-pressure gradient between the abdomen and thorax. This can lead to the herniation of the lower esophageal sphincter into the thorax, impairing its barrier function. Consequently, this may allow increased gastric reflux in asthmatics with hyperinflation (24).

Additionally, certain asthma medications attempting to reduce hyperinflation may worsen acid reflux. β-agonists have been shown to reduce the tone of the lower esophageal sphincter in a dose-dependent manner (25). This raises the possibility of a vicious cycle where asthma symptoms caused by GERD lead to the increased use of bronchodilators, exacerbating GERD symptoms.

Other pathways may shed light on the potential mechanisms by which GERD may cause or exacerbate airway obstruction in patients with asthma. Firstly, repetitive GERD-related acid micro-aspiration into the larynx and upper airways can exacerbate respiratory conditions, including asthma and chronic obstructive pulmonary disease, according to a previous histological lung analysis in a rat model of surgically-induced GERD (26). Secondly, reflux episodes can trigger an esophago-bronchial vagal reflex and increased bronchial reactivity, potentially leading to bronchoconstriction. A previous study involving intra-esophageal acid infusions demonstrated a decrease in peak expiratory flow even without signs of micro-aspiration, implying vagally-mediated reflex involvement (27). Of note, these broncho-constrictive reactions were not dependent on esophageal mucosal inflammation or micro-aspiration. These molecular findings clarify the complex link between asthma and GERD induced by HH.

Using the GINA assessment of asthma control, the present study revealed distinct trends in the severity of asthma among the study participants. Specifically, 23.88% had ‘well-controlled’ asthma symptoms, 34.33% had ‘partially controlled’ asthma, and 41.79% had ‘uncontrolled’ asthma symptoms. This distribution is in contrast with the results of the PRISMA study (28), a large cross-sectional study involving 2,853 adults with asthma, where up to 64.4% of patients had controlled asthma, 15.8% had partially controlled asthma and 19.8% had uncontrolled asthma. Similarly, another recent study conducted in the general population (29), reported that uncontrolled asthma was present in 26% and partially controlled asthma in 22.9% of patients.

In the present study, it was reported that patients with HH appeared to have a higher prevalence of ‘uncontrolled’ and ‘partially controlled’ asthma. This may be due to the potential impact of HH on respiratory function, leading to an increased asthma severity, or underlying common mechanisms contributing to both conditions.

In the present study, the frequency of respiratory symptoms (outside of an asthma diagnosis) was reported using a previously published questionnaire that evaluated the status of occupational asthma in a cohort of workers (30). The findings obtained herein indicated notable differences between patients diagnosed with HH and those with untreated asthma-like symptoms described in the worker cohort in the previous study (30). Chest wheezing (13.74 vs. 26.20%) and waking up due to coughing (13.99 vs. 51.50%) were less common in the population of patients with HH compared with the worker population. However, the patients in the present study reported a higher prevalence of symptoms, such as waking up due to shortness of breath (22.54 vs. 11.20%) and asthma attacks (1.81 vs. 0.6%). These differences might be due to inherent bias in the study sample selection. The overestimation of respiratory symptoms in the worker population may be attributed to variations in age and occupational exposure. Specifically, the worker population was younger than the average age of 67 years reported in the present study and environmental factors specific to the workplace could play a role in exacerbating respiratory symptoms.

The evaluation of GERD symptoms and asthma among patients with HH indicated a higher rate of asthma among patients with GERD symptoms (16.85%) compared to those without (11.49%); however, this difference did not reach statistical significance (P-value=0.1). In a previous study on >100,000 patients, patients with GERD were 1.51-fold (1.43-1.59) more likely to suffer from asthma than patients without GERD (31).

Patients undergoing treatment for GERD with PPIs or other anti-reflux medications had a higher prevalence of asthma (17.48%) than those not receiving anti-acid treatment (10.18%), with a statistically significant P-value of 0.03. This aligns with the findings of a broader research (32), where the overall incidence of asthma was 1.58-fold higher in patients treated with PPIs than those not receiving PPIs.

While the present study did not detect a significant sex predilection for asthma presentation in patients with HH, previous studies have suggested that in adulthood, females exhibit increased an prevalence and severity of asthma (33-35).

In order to manage their GERD symptoms, patients with HH are frequently advised to make certain lifestyle changes, such as losing weight, changing their diet and sleeping at an angle. Controlling asthma may be indirectly affected by these lifestyle modifications. For example, losing weight can help asthmatic outcomes by enhancing lung function and decreasing systemic inflammation. However, the stress of dealing with several chronic illnesses (HH, asthma and GERD) may cause patients to adhere to these lifestyle treatments less than optimally, particularly if they prioritize one ailment over the others.

Finally, it is important to recognize the psychological toll that having both asthma and HH can have. The symptoms of both conditions, fatigue, chest discomfort and shortness of breath, can overlap and result in severe anxiety, which may lead to a psychosomatic amplification of symptoms and worsen asthma severity, as well as the perceived impact of HH on daily life. Including mental health services in the treatment plan for these patients may help lessen this burden and enhance symptom control.

In summary, although there are clear physiological connections between HH and asthma, patients with both conditions require a comprehensive approach to care that takes into account not only pharmacological interventions, but also lifestyle, psychosocial and even neurogenic aspects. The knowledge gained from this research opens the door to more complex, interdisciplinary approaches to treatment, guaranteeing that patients get all-encompassing assistance for both their digestive and respiratory systems.

As regards, limitations and bias, firstly, the present study relied on a retrospective design, which may have introduced biases related to data collection. A limitation of the present study is the variability in patient data collection over time, as the health status of patients from earlier years, such as 2020, may have evolved by the final analysis in 2023. Disease progression in conditions such as HH and asthma could mean that more recent data could better reflect current health trends. Additionally, in the absence of a control group (patients without HH evaluated for asthma or other respiratory conditions), a direct association between the prevalence of asthma and the presence of HH cannot be established. Comparisons had to be primarily made with data from the literature. In addition, the present study did not stratify the results based on potential confounders, such as comorbidities, age and smoking status; which limits the generalizability of the results.

In conclusion, the present study uniquely identifies a significantly higher prevalence of asthma (14.79%) among patients with HH compared to global asthma prevalence rates. Notably, 41.79% of asthmatic patients demonstrated an inadequate control of their condition, indicating that asthma severity in this population may be underrecognized and undertreated. The findings from the present study suggest that HH may play an underappreciated role in respiratory health, warranting increased clinical vigilance and a proactive approach to diagnosing and managing respiratory comorbidities in this population.

In clinical practice, while the presence of HH should be considered when evaluating patients with difficult-to-control asthma, further evidence is required to support their routine identification as a key factor in asthma management. Longitudinal studies with more robust data and extended follow-up are necessary to confirm the potential progression of asthma in patients with HH and to evaluate the impact of specific interventions on respiratory outcomes. Additionally, more detailed research into the underlying mechanisms linking HH and asthma may shed light on potential pathways for targeted therapies and refined treatment approaches for these patients.

## Supplementary Material

Questionnaires and permissions

## Figures and Tables

**Table I:** Demographic profile of the study population.

Demographic characteristics	Total (n=453)
Age, years (median; Q1-Q3)	67 (63-83)
Sex, n (%)	
Males	173 (38.19%)
Females	280 (61.81%)

**Table II:** Control of asthma according to GINA asthma symptom control.

Asthma control	Score	No. of patients	Total no. of patients	Percentage
Controlled asthma	Score 0	16	16	23.88
Partially controlled asthma	Score 1	10	23	34.33
	Score 2	13		
Uncontrolled asthma	Score 3	17	28	41.79
	Score 4	11		

GINA, Global Initiative for Asthma.

**Table III:** Percentage of asthma-related respiratory symptoms according to ECRHS.

Symptom	No. of patients	Percentage
Asthma attacks	7	1.81
Chest wheezing	52	13.47
Shortness of breath with wheezing	22	5.7
Waking up with a feeling of tightness or shortness of breath	87	22.54
Waking up due to a coughing fit	54	13.99
Symptoms and score		
Score 0/5	250	64.77
Score 1/5	82	21.24
Score 2/5	30	7.77
Score 3/5	16	4.15
Score 4/5	8	2.07
Score 5/5	0	0.00
Average no. per subject	0.8	

ECRHS, European Community Respiratory Health Survey.

**Table IV:** Association of GERD symptoms, medical treatment and sex with the prevalence of asthma.

	Known to have asthma		
Variables	Yes (%)	No (%)	P-value
Symptoms of GERD, n (%)			0.1
Present	47 (16.85)	232 (83.15)	
Absent	20 (11.49)	154 (88.51)	
Medical treatment of GERD, n (%)			0.03
Present	50 (17.48)	236 (82.52)	
Absent	17 (10.18)	150 (89.82)	
Sex, n (%)			0.07
Male	19 (10.98)	154 (89.02)	
Female	48 (17.14)	232 (82.86)	

GERD, gastroesophageal reflux disease.

## Data Availability

The datasets used and/or analyzed during the current study are available from the corresponding author on reasonable request.

## Authors' information

The ORCID IDs of the authors are as follows: Michel Abou Khalil: 0009-0007-2038-7678; Zeina Aoun Bacha: 0000-0001-9723-1267; Hind Eid: 0000-0002-9306-4040; Moussa Riachy: 0000-0003-2146-6945.
