# Supplementary material for: Elevated frequency and severity of asthma in patients with hiatal hernia: A retrospective study
Source: Med Int (Lond). 2024 Dec 2;5(1):10. doi: 10.3892/mi.2024.209 (PMC11668131; doi:10.3892/mi.2024.209)
Supplement: Questionnaires and permissions [file Supplementary_Data.pdf]

Questionnaires and permissions

Hiatal Hernia		
**1. Do you have symptoms of gastroesophageal reflux (heartburn, unexplained cough, sensation of a lump in the throat…)?**		
**2. Are you taking medication for your hiatal hernia? (If yes, please specify whether the treatment is aimed at managing reflux symptoms or providing gastric protection in the context of polypharmacy.)**		
**3. Have you undergone surgery for your hiatal hernia?**		

eTable 1: Patient Questionnaire on Hiatal Hernia Symptoms, Medication, and Surgical History

Asthma (In the past 12 months)		
**4. Are you diagnosed and actively treated by a pulmonologist for asthma?**		
**If yes, proceed to question 5.**		
**If no, proceed to question 9.**		
**5. Do you have daytime asthma symptoms more than twice a week?**		
**6. Do you experience nighttime awakenings due to asthma?**		
**7. Do you use a SABA inhaler for symptom relief more than twice a week?**		
**8. Do you have any activity limitations due to asthma?**		
**9. Have you had a wheezing episode or a whistling sound in your chest?**		
**10. Have you been short of breath in the presence of wheezing?**		
**11. Have you woken up with a feeling of tightness or shortness of breath in your chest?**		
**12. Have you been awakened by a coughing fit?**		
**13. Have you had an asthma attack?**		
**14. Are you currently taking medication (including inhalers, aerosols, or tablets) for asthma?**		
**15. Do you have nasal allergies, including hay fever?**		

eTable 2: Asthma Assessment: GINA Symptom Control and ECRHS Respiratory Health Questionnaire

Questions 5 to 8 are extracted from the GINA Asthma Symptom Control questionnaire, and questions 9 to 15 are extracted from the European Community Respiratory Health Survey (ECRHS). Both are validated questionnaires and do not require copyright permissions for use.
